# A systems biology approach to understand temporal evolution of silver nanoparticle toxicity

**DOI:** 10.1038/s41540-025-00561-7

**Published:** 2025-07-19

**Authors:** Seung-Geun Park, Eunseo Lee, Hyun-Yi Kim, Tae Hyun Yoon

**Affiliations:** 1 Department of Chemistry, Hanyang University, Seoul, Republic of Korea; 2 NGeneS Inc., Ansan-si, Republic of Korea; 3 Yoon Idea Lab. Co. Ltd, Seoul, Republic of Korea; 4 Research Institute for Convergence of Basic Science, Hanyang University, Seoul, Republic of Korea; 5 Institute of Next Generation Material Design, Hanyang University, Seoul, Republic of Korea

**Keywords:** Cell biology, Computational biology and bioinformatics, Molecular biology, Systems biology

## Abstract

Silver nanoparticles (AgNPs) are widely used in industrial and biomedical applications, however, their toxicity mechanisms at the molecular level are not completely understood. To address this gap, we investigate the temporal dynamics of gene expression in human lung epithelial cells exposed to AgNPs, integrating transcriptomic analysis, gene ontology (GO) enrichment, protein-protein interaction (PPI) networks, and dynamic simulations. GO analysis highlights early activation of ribosomal biogenesis and stress pathways, transitioning DNA repair and cell cycle regulation at later stages. PPI networks identify ribosomal proteins and DNA damage regulators as key hub genes. Dynamic simulations modeled gene expression changes over 48 hours, uncovering sequential activation of stress response genes, followed by DNA repair attempts and apoptotic signaling as cellular damage persisted. Through modeling the interplay between molecular responses and cell viability, the simulations provided a predictive temporal framework for advancing nanotoxicology research, providing insights into AgNPs-induced molecular disturbances, contributing to safety assessments.

## Introduction

Nanotechnology, the manipulation and control of matter at the nanometer scale (10^−9^), has revolutionized various fields by enabling the development of materials with unique physical and chemical properties^1,2^. Among these, silver nanoparticles (AgNPs) have gained considerable attention due to their distinctive antimicrobial, optical, and electrical properties^3–5^, making them highly valuable in medical applications^3^, environmental systems^6^, and industrial processes. Despite these advantages, increasing concerns have emerged regarding their potential adverse effects on biological systems, particularly their toxicity. Several studies have demonstrated that AgNPs can generate reactive oxygen species (ROS), leading to oxidative stress, DNA damage, and apoptosis^7–13^. Furthermore, prolonged exposure to AgNPs has been associated with respiratory disorders in humans, highlighting the potential risks to human health^14^.

However, the precise molecular mechanisms driving these effects are still insufficiently understood, especially the temporal evolution of these toxicological responses^15,16^. While previous research has established that AgNPs can cause oxidative stress and disrupt key cellular functions such as cytoskeletal stability^17^, and mitochondrial energy production^18^, these studies have largely focused on static endpoints, capturing only a snapshot of cellular changes. Although research has identified AgNPs-induced ROS generation and subsequent cell death^19–21^, it remains unclear how these processes unfold over time and how different molecular pathways are sequentially activated or inhibited. Understanding the temporal progression of cellular responses is essential to fully grasp the mechanisms of toxicity. This knowledge enables the identification of critical time points for targeted safety interventions to predict and mitigate risks.

The primary objective of this study is to investigate the temporal changes in gene expression under AgNPs exposure and to elucidate the molecular mechanisms of AgNPs-induced toxicity. By examining these changes over time, we aim to provide a dynamic view of how AgNPs interact with biological systems at the molecular level. Specifically, we will focus on identifying key transcriptomes and pathways involved in oxidative stress, endoplasmic reticulum (ER) stress, DNA damage, and apoptosis, which are crucial to the toxicological effects of AgNPs. In doing so, we aim to bridge the gap between static molecular observations and the dynamic processes that regulate nanoparticle-induced cellular stress.

To achieve these goals, we utilized publicly available transcriptomic datasets containing more than two time points to identify differentially expressed genes (DEGs) in response to AgNPs. This will allow us to capture the molecular changes at various time points, offering insights into the sequence of gene activation or inhibition. Additionally, we constructed protein-protein interaction (PPI) networks using the Search Tool for the Retrieval of Interacting Proteins (STRING)^22^ to determine critical hub genes and pathways affected by nanoparticle-induced stress. STRING is particularly well-suited for this analysis, as it integrates known and predicted protein interactions, allowing us to map out key molecular networks. Furthermore, we used CellDesigner^23,24^ to visualize these interactions and COPASI^25^ to estimate rate constants from rate equations and perform dynamic simulations. It provides a comprehensive view of how gene expression evolves over time under AgNPs-induced stress, enabling us to explore both static molecular interactions and dynamic processes.

The significance of this research lies in its potential to advance the understanding of nanotoxicology by providing a detailed temporal framework for nanoparticle-induced molecular disturbances. By tracking how gene and protein expression changes over time, this study aims to contribute to more accurate safety assessments and predictive models for the use of nanoparticles in medical and industrial applications. Ultimately, the insights gained from this research could guide the development of safer nanomaterials and inform regulatory guidelines for their use.

## Results

### Identification of DEGs in response to AgNPs exposure

**Fig. 1:**
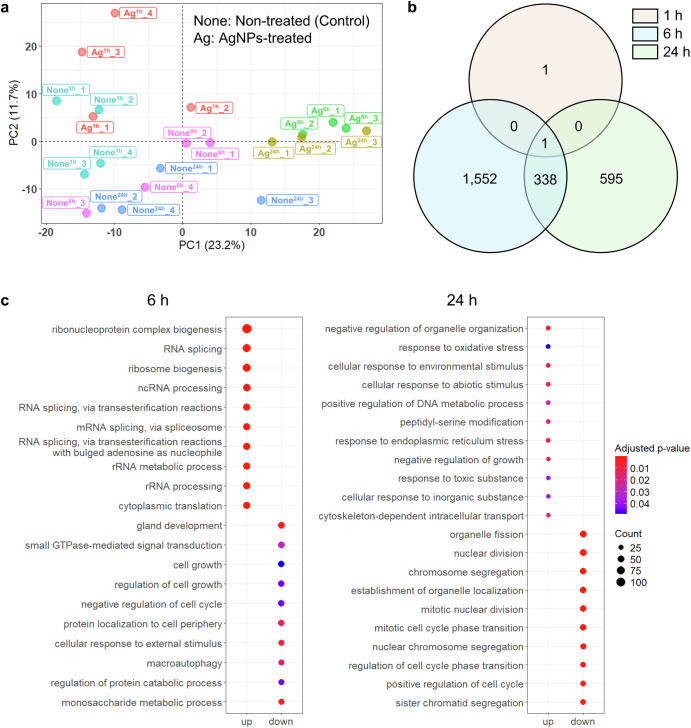
Impact of AgNPs treatment on cellular responses over time. **a** Principal component analysis (PCA) of gene expression profiles (four biological replicates each; for treated group of 6 h and 24 h, three biological replicates each). PCA plot showing the clustering of gene expression profiles from cells treated with AgNPs and untreated controls at different time points (1 h, 6 h, and 24 h). Each point represents an individual sample, with samples grouped by treatment and time point as indicated by color. **b** Venn diagram of differentially expressed genes (DEGs). Venn diagram displays the overlap of DEGs across three time points (1 h, 6 h, and 24 h) following AgNPs treatment. Each circle represents the set of DEGs at a specific time point, highlighting both unique and common gene expression responses over time. DEGs were labeled as genes for which the Benjamini–Hochberg adjusted *p*-value was less than 0.05. **c** Gene ontology (GO) enrichment analysis of upregulated and downregulated genes at 6 h and 24 h following AgNPs treatment. Dot plots depicting the GO terms enriched among upregulated (left columns in each plot) and downregulated (right columns in each plot) genes at 6 h (left panel) and 24 h (right panel) post-treatment with AgNPs. Each dot represents a GO term, with the color indicating the adjusted *p*-value (red signifies higher significance) and the dot size corresponding to the number of genes associated with each term (larger dots represent a greater gene count). Benjamini–Hochberg adjusted *p*-values < 0.05 were considered statistically significant.

To assess the cellular response to AgNPs over time, transcriptomic analyses were conducted at multiple time points post-exposure. Figure 1a shows a Principal Component Analysis (PCA) plot of gene expression profiles in treated and control cells at 1, 6, and 24 h post-exposure. The PCA indicated distinct clustering patterns between treated and control samples, with clear separation of time points along the principal components. PC1 and PC2 accounted for 23.2% and 17.7% of the variance, respectively, illustrating a temporal shift in gene expression following AgNPs treatment. To further confirm the robustness of these clustering patterns, we applied t-SNE and UMAP for dimensionality reduction, both of which reproduced the clear separation between treated and control samples initially observed in the PCA (Supplementary Fig. S1a, b).

DEGs were identified at 1, 6 and 24 h (Fig. 1b, Supplementary Data 1). Only minimal transcriptional activity was observed at 1 h, with limited DEGs (1 upregulated gene, 1 downregulated gene), suggesting an initial cellular tolerance to AgNPs exposure. Due to the lack of significantly regulated genes at this early time point, 1 h data were not included in the gene ontology (GO) analysis (Fig. 1c). However, a substantial transcriptional response was evident by 6 h, with 854 genes upregulated and 1037 genes downregulated, reflecting a robust cellular stress response. At 24 h, the number of DEGs decreased, indicating a partial adaptation or stabilization in gene expression profiles, with 376 genes upregulated and 558 genes downregulated. This temporal pattern implies a dynamic transcriptional response to AgNPs, peaking at 6 h before a potential recovery phase.

### Enrichment analysis of biological processes affected by AgNPs exposure

To explore the cellular pathways affected by AgNPs exposure, we conducted GO enrichment analysis on DEGs identified at 6 and 24 h (Fig. 1c, Supplementary Data 2). At 6 h, the top enriched GO terms among upregulated genes included processes related to ribonucleoprotein complex biogenesis, RNA splicing, and ribosome biogenesis, alongside pathways associated with protein folding. This enrichment suggests that early AgNPs exposure primarily impacts protein synthesis machinery and cellular stress responses. In contrast, at 24 h, the enriched processes shifted towards pathways involved in oxidative stress responses, cellular response to toxic substances, and DNA biosynthetic processes. Additionally, the GO analysis highlighted mitotic nuclear division and chromosome segregation as significantly enriched processes at this later stage, indicating a shift towards cell cycle regulation and potential recovery mechanisms.

### Protein-protein interaction network and key hub genes

**Fig. 2:**
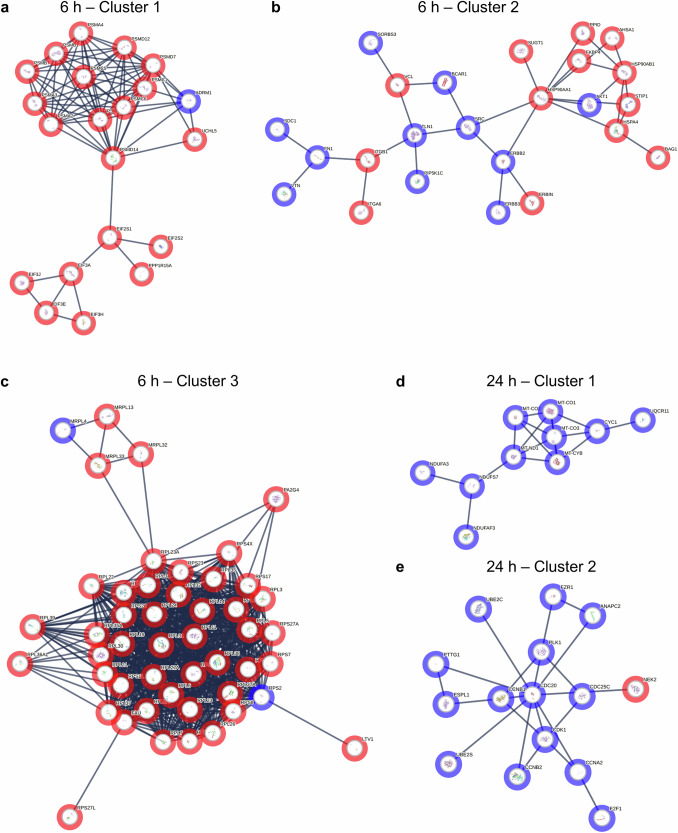
PPI networks of DEGs at 6 and 24 h exposure to AgNPs. **a**–**c** Clusters identified in the PPI network at 6 h, representing early-stage responses to AgNPs-induced stress. **d**, **e** Clusters identified in the PPI network at 24 h, showing changes in gene interactions and adaptations over prolonged exposure. Red nodes represent upregulated genes, while blue nodes represent downregulated genes. The size of the node corresponds to the degree of interaction, with larger nodes representing hub genes. It presents selected clusters from the overall PPI network, highlighting distinct network structures and interaction patterns at each time point.

To elucidate the molecular interactions underlying the cellular response to AgNPs exposure, we generated PPI networks at 6 and 24 h (Fig. 2a–e, Supplementary Data 3). At 6 h, the PPI network revealed a high concentration of ribosomal and stress-related genes as central nodes. Key hub genes such as *RPS27A*, *RPS11*, and *RPL23A* exhibited the highest interaction degrees (Table 1), highlighting their significant roles in early cellular responses. These ribosomal genes are involved in processes associated with protein synthesis and cellular stress management. It suggests that the immediate impact of AgNPs focuses on enhancing protein production machinery to counteract initial stress. The focus of the PPI network shifted at 24 h, with genes involved in cell cycle regulation and DNA damage response becoming predominant. At this later time point, genes such as *CDC20*, *CDK1*, and *PLK1* emerged as key hubs, characterized by high degrees of interaction (Table 1). This temporal transition from ribosomal to cell cycle-related hub genes aligns with the observed adaptive response. In other words, the initial upregulation of ribosomal activity for protein synthesis is followed by regulatory mechanisms for cell survival and genome stability under prolonged nanoparticle exposure.

**Table 1:** List of key hub genes from PPI network analysis at 6 and 24 h

Condition	Gene symbol	Degree	log_2_FC	Adjusted *p*-value
6 h	*RPL23A*	43	0.29	2.91E−02
	*RPL19*	40	0.51	7.77E−04
	*RPS11*	40	0.32	1.20E−02
	*RPL21*	40	0.58	6.92E−04
	*RPL30*	40	0.38	1.77E−03
	*FAU*	40	0.30	2.85E−02
	*RPL23*	39	0.74	1.42E−07
	*RPS24*	39	0.67	2.32E−07
	*RPL5*	39	0.55	5.41E−07
	*RPL24*	39	0.58	1.89E−06
24 h	*CDC20*	10	−0.87	1.39E−07
	*COX1*	5	−0.63	3.85E−03
	*CYTB*	5	−0.51	4.65E−02
	*ND1*	5	−0.82	4.05E−04
	*CDK1*	5	−0.51	2.27E−02
	*COX3*	5	−0.74	9.95E−04
	*CCNB1*	4	−1.15	8.48E−13
	*PLK1*	4	−1.89	3.17E−37
	*CYC1*	4	−0.39	1.92E−02
	*COX2*	4	−0.59	1.04E−02

Genes are ranked by their degree and adjusted *p*-value. In cases where genes had the same degree, they are ordered by adjusted *p*-value, with lower values prioritized.

These findings underscore a distinct temporal adaptation within the cellular response to AgNPs toxicity, where initial activation of ribosomal pathways gives way to cell cycle control mechanisms as exposure persists. The identification of these time-dependent hub genes provides insights into the sequential activation of stress response pathways, illustrating how cells dynamically reprogram their molecular interactions in response to prolonged exposure to silver nanoparticles.

### Molecular pathway of AgNPs-induced cellular stress and apoptosis responses

Previous studies have shown that exposure to nanoparticles, including AgNPs, initiates a multi-phased cellular stress response. Initially, oxidative stress is triggered as ROS levels increase, activating antioxidant pathways that mitigate ROS-related damage^26^. In response to prolonged stress, ER homeostasis may be disrupted, leading to unfolded protein response (UPR) activation as cells manage increased protein-folding demands^27,28^. Furthermore, the accumulation of DNA damage initiates DNA repair pathways, primarily regulated by p53 and ATM/ATR proteins, which are critical for genomic stability^29–31^. Apoptotic pathways are finally triggered to remove irreversibly damaged cells as cellular damage continues beyond repairable levels^32^.

**Fig. 3:**
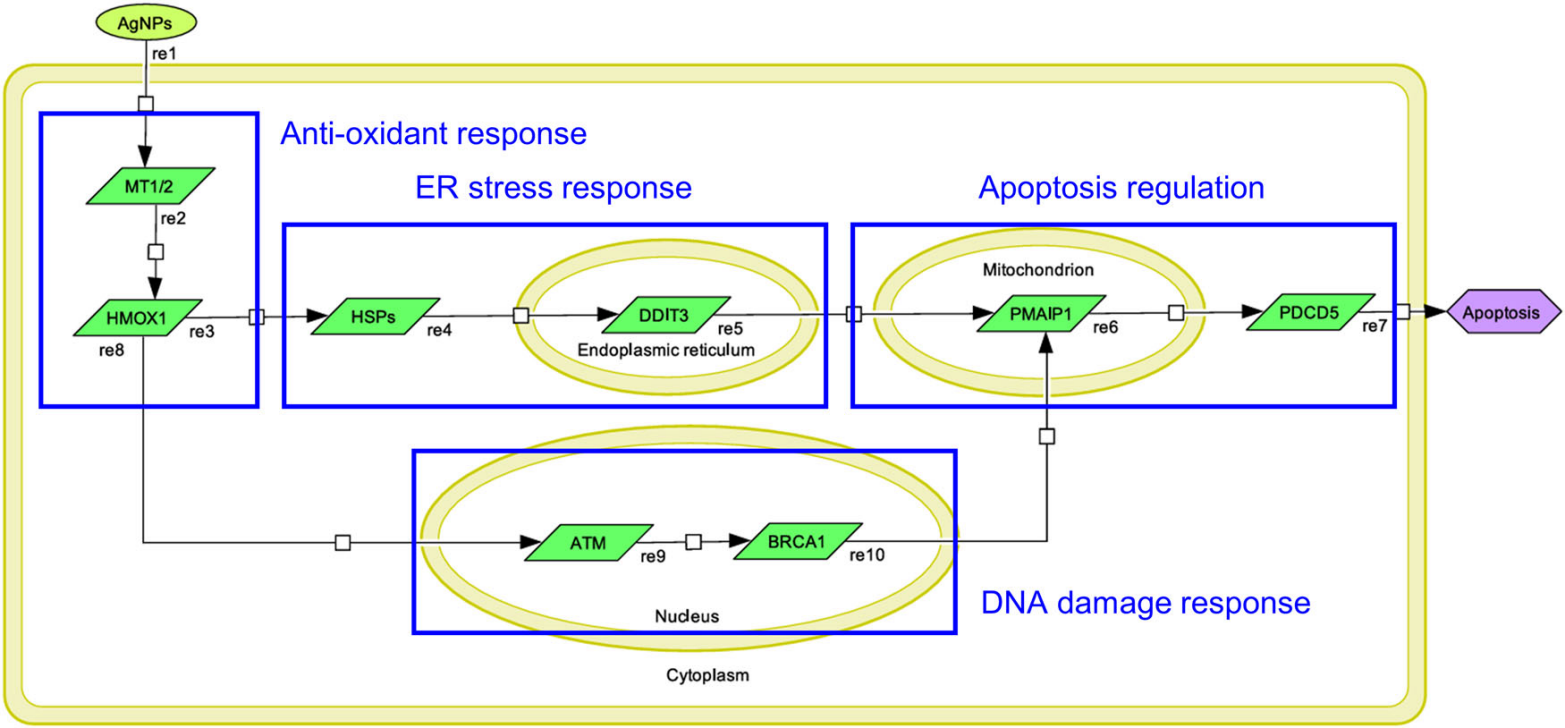
Pathway map of AgNPs-induced cellular stress and apoptotic pathways. A schematic illustration of the cellular pathways involved in the response of AgNPs exposure, highlighting key stress responses and apoptotic signaling mechanisms. The exposed substance (AgNPs) is shown in lime green, RNAs are shown in green and cellular events are shown in purple.

We constructed a pathway map with CellDesigner based on DEG analysis, functional annotations, and a comprehensive literature review. The pathway map outlines the sequential responses to AgNPs-induced stress, focusing on the transcriptional regulation of mRNA (Fig. 3). It features two main branches, each highlighting the flow of events involving specific genes and their roles in cellular responses. The first branch begins with the activation of Metallothionein 1/2 (*MT1* and *MT2*), triggering the transcription of *HMOX1* via stress-induced signaling. This activation sets off a cascade that includes the upregulation of heat shock proteins (HSPs), which play a crucial role in mitigating protein misfolding during ER stress. Following this, *DDIT3* is upregulated, facilitating either ER stress resolution or apoptosis depending on the severity of the damage. This pathway ultimately concludes with the activation of *PMAIP1* and *PDCD5*, which drive apoptotic signaling. The second branch shares an initial activation of *MT1/2* and *HMOX1* but diverges as it transitions to mechanisms focused on DNA damage repair. Central to this response is the activation of *ATM*, a key regulator of the DNA damage response, followed by the involvement of *BRCA1*, which plays a pivotal role in maintaining genomic stability. When repair processes are insufficient to resolve the damage, this pathway converges with the first branch, culminating in the activation of *PMAIP1* and *PDCD5* to initiate apoptosis.

### Kinetic modeling of gene interactions in response to AgNPs exposure

Kinetic modeling was performed to quantify the interactions between key genes involved in the cellular stress response to AgNPs exposure based on the molecular pathway map. Under the assumption of mass action kinetics, all reactions were considered irreversible, and gene concentrations were represented as normalized read counts derived from transcriptomic datasets.

Parameter estimation was conducted using COPASI, a biochemical simulation software, which calculated reaction rate constants based on observed time-course gene expression data. Initial gene expression values were set using normalized counts at control sample (0 h). The model was iteratively fitted to the temporal dynamics of gene expression across multiple time points by minimizing deviations between observed and simulated levels. 10 reactions were modeled, each described by a kinetic law and its corresponding estimated rate constant (Table 2). These reactions encompass oxidative stress responses, ER stress pathways, DNA damage repair mechanisms, and apoptotic signaling. The estimated rate constants ranged from strongly positive (e.g., re5, *k* = 0.926291) to negative (e.g., re8, *k* = −0.0367482), reflecting dynamic patterns of activation and inhibition among molecular components.

**Table 2:** Kinetic reactions and estimated rate constants for modeling gene interactions under AgNPs-induced stress

Reactions	Kinetics	Estimated rate constant (h^−1^)
re1	${k}_{1}\left[{AgNPs}\right]=[{MT}1/2]$	0.67147
re2	${k}_{2}\left[{MT}1/2\right]=[{HMOX}1]$	0.0267081
re3	${k}_{3}\left[{HMOX}1\right]=[{HSPs}]$	0.0330709
re4	${k}_{4}\left[{HSPs}\right]=[{DDIT}3]$	0.0280639
re5	${k}_{5}\left[{DDIT}3\right]=[{PMAIP}1]$	0.926291
re6	${k}_{6}\left[{PMAIP}1\right]=[{PDCD}5]$	0.000608018
re7	${k}_{7}\left[{PDCD}5\right]=[{Cell\; viability}]$	−0.012447
re8	${k}_{8}\left[{HMOX}1\right]=[{ATM}]$	−0.0367482
re9	${k}_{9}\left[{ATM}\right]=[{BRCA}1]$	−0.223519
re10	${k}_{10}\left[{BRCA}1\right]=[{PMAIP}1]$	−0.105018

Note that positive rate constants indicate activation, whereas negative values represent inhibitory interactions.

### Dynamic simulation of temporal gene expression in AgNPs-induced stress response

Building on the kinetic modeling framework, we performed dynamic biochemical simulations using COPASI to model the temporal progression of gene expression levels under AgNPs-induced stress. Initial expression levels for the key genes included in the model are listed in Table 3.

**Table 3:** Initial expression levels of species included in the dynamic simulation

Species	Initial expression level	Categories
AgNPs	$38.6$	Initial point
*MT1/2*	16.3609	Anti-oxidant response
*HMOX1*	200.014	Anti-oxidant response
*HSPs*	556.254	ER stress response
*DDIT3*	7.40356	ER stress response
*PMAIP1*	22.9312	Apoptosis progression
*PDCD5*	128.955	Apoptosis progression
*ATM*	35.7379	DNA damage response
*BRCA1*	97.139	DNA damage response
Cell viability	100	End point

**Fig. 4:**
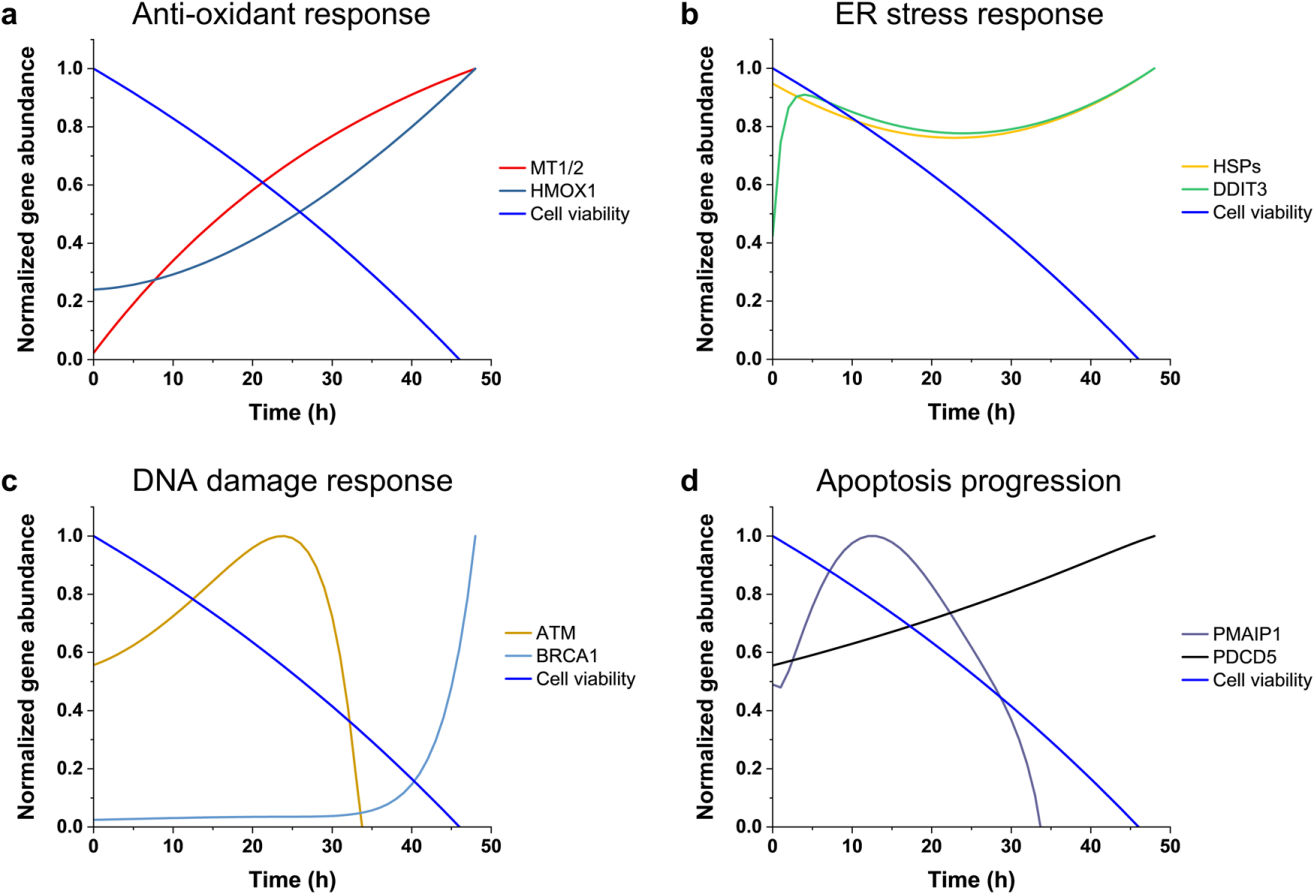
Simulation of temporal gene expression changes under AgNPs-induced stress. **a** Anti-oxidant response, showing expression changes in *MT1/2* and *HMOX1*, alongside cell viability. **b** ER stress response, highlighting *HSPs* and *DDIT3* expression dynamics. **c** DNA damage response, focusing on *ATM* and *BRCA1*. **d** Apoptosis progression, illustrating changes in *PMAIP1* and *PDCD5* expression. Gene expression levels were normalized by dividing the observed values at each time point by the maximum value within the dataset for that gene, resulting in values ranging from 0 to 1. The simulation was conducted using dynamic biochemical models based on observed transcriptomic data, capturing the progression of cellular stress responses.

Exposure to AgNPs resulted in a continuous decline in cell viability throughout the time course (Fig. 4a–d). The observed gene expression patterns revealed distinct changes associated with key stress responses and apoptotic signaling. In the antioxidant response, *MT1/*2 expression showed a logarithmic increase, while *HMOX1* expression exhibited an exponential increase (Fig. 4a). For the ER stress response, HSPs expression decreased initially, reaching a minimum at 23 h before increasing steadily. *DDIT3* expression sharply increased within the first 4 h, then slightly decreased, followed by another increase starting at 24 h (Fig. 4b). In the DNA damage response, *ATM* expression increased, peaking at 23 h, then sharply decreased and became depleted by 34 h. *BRCA1* expression increased modestly early on but showed a marked rise starting at 27 h (Fig. 4c). Apoptotic signaling was characterized by changes in *PMAIP1* and *PDCD5* expression. *PMAIP1* expression increased early, peaked at 13 h, then declined and became depleted by 34 h. In contrast, *PDCD5* expression exhibited a consistent increase throughout the time course (Fig. 4d).

## Discussion

In this study, we explored the temporal dynamics of gene expression in response to AgNPs exposure using publicly available transcriptomic datasets. Our findings reveal the sequential activation of molecular pathways associated with oxidative stress, DNA damage, and apoptosis, offering valuable insights into the mechanisms underlying AgNPs-induced toxicity. While the previous study provided a robust foundation for understanding the molecular responses to AgNPs exposure, their analysis primarily focused on identifying static molecular changes at discrete time points^33^. In contrast, our study moves beyond static endpoint studies by introducing a temporal perspective, modeling the sequential activation and progression of key stress pathways over 48 h. This dynamic approach highlights not only the transient and adaptive nature of cellular responses but also provides a predictive framework for understanding how cellular stress progresses to apoptosis under prolonged nanoparticle exposure. To further explore these temporal molecular dynamics, we analyzed the time-resolved transcriptional activity across key pathways.

The cellular response was characterized by significant transcriptional activity, with over 1800 DEGs at 6 h. Enriched pathways during this early phase were dominated by processes related to ribosomal biogenesis and protein folding, reflecting an acute stress response targeting the protein synthesis. AgNPs have been found to interact with ribosomal subunits, particularly affecting the transcription and translation processes^34^. This mechanism can lead to the formation of immature precursor proteins and disrupt normal protein production pathways in cells. It may appear to represent an early cellular stress response to nanoparticle exposure, where cells attempt to increase their protein synthesis to mitigate the stress. Hub genes such as *RPS27A* and *RPS11* play pivotal roles in these processes, suggesting that ribosomal proteins are central to the initial response to AgNPs exposure. The number of DEGs decreased to 934 at 24 h, suggesting a shift in cellular dynamics from acute stress to adaptive responses. Functional enrichment analyses revealed a focus on oxidative stress mitigation, DNA repair mechanisms, and cell cycle regulation. Key genes such as *CDK1* and *CDC20*, involved in mitotic progression and DNA damage repair, were central players during this phase. These findings suggest that while the initial exposure to AgNPs induces robust cellular stress, prolonged exposure may activate recovery mechanisms aimed at restoring homeostasis.

However, these responses appear insufficient to fully counteract the sustained toxicity, as indicated by the continued decline in cell viability. Simulation-based analysis further elucidates the sequential activation and interplay of oxidative stress, ER stress, DNA damage, and apoptotic pathways over time. The simulation highlights the dynamic engagement of *MT1/2* as the first line of defense, which mitigates metal-induced oxidative stress by binding silver ions (Ag⁺) and regulating redox balance. This response triggers downstream transcriptional activation through metal-responsive transcription factor-1 (MTF-1), amplifying oxidative defense^35–38^. The subsequent upregulation of *HMOX1* reflects a secondary phase of defense, focused on heme metabolism and ROS detoxification^39–41^. Despite these efforts, the prolonged oxidative damage leads to a tipping point where protective mechanisms are overwhelmed. In the context of ER stress, the transcriptional patterns of *HSPs* and *DDIT3* illustrate the transition from adaptive stress responses to apoptosis. The initial suppression of *HSPs* mRNA levels suggests an overload of protein folding system, followed by later upregulation aimed at restoring ER function. Conversely, the biphasic activation of *DDIT3* represents a decisive shift toward apoptosis^28,42^, with its reactivation at 24 h indicating a failure to manage persistent stress. Simulation data also emphasizes the dynamic progression of DNA damage responses. The early upregulation of *ATM* suggests an immediate effort to detect and repair DNA lesions, but its subsequent depletion may reflect the collapse of repair pathways. The delayed activation of *BRCA1* indicates ongoing attempts to maintain genomic stability, yet the failure to fully resolve damage drives the activation of apoptotic pathways. The sequential activation of apoptotic markers, including *PMAIP1* and *PDCD5*, further validates this pathway. *PMAIP1* facilitates mitochondrial outer membrane permeabilization (MOMP) in the early apoptotic phase^43,44^, while the sustained upregulation of *PDCD5* amplifies pro-apoptotic signals, ensuring commitment to programmed cell death^45,46^. These findings are consistent with the observed decline in cell viability, highlighting the culmination of prolonged stress responses into apoptosis.

Our study provides a comprehensive understanding of the molecular dynamics underlying AgNPs-induced toxicity by integrating DEG analysis, pathway enrichment, PPI networks, and temporal simulations. The distinct temporal patterns of oxidative stress, ER stress, and DNA damage responses, coupled with their eventual progression to apoptosis, underscore the limitations of cellular recovery under sustained nanoparticle exposure. While this study provides significant insights into the temporal dynamics of AgNPs-induced cellular responses, it is important to acknowledge its limitations. First, the simulation framework assumes that all reactions follow mass action kinetics and are irreversible. While this assumption simplifies model construction and interpretation, it has not been empirically validated in the context of AgNPs-induced cellular responses. Future studies should address these limitations by incorporating reversible reaction dynamics and experimentally validating key reaction rates to enhance biological relevance of the model. Second, the reliance on RNA-seq data presents inherent limitations. RNA-seq captures transcriptional changes, but these do not always correlate directly with functional protein levels due to additional regulatory processes such as miRNA-mediated control and protein degradation^47,48^. Moreover, it fails to detect post-translational modifications (PTMs), which are crucial for determining protein activity, stability, and progression of signaling pathway^29,49,50^. Critical processes such as oxidative stress responses and apoptosis often depend heavily on PTMs like phosphorylation or ubiquitination, which RNA-seq cannot detect. To achieve a more comprehensive understanding of molecular mechanisms, future work should integrate proteomic and metabolomic data with transcriptomic analyses. For instance, proteomic datasets for key stress markers (e.g., MT1/2, HO-1, DDIT3) and metabolomic profiles of associated small molecules can be generated and combined with transcriptomic profiles via Multi-Omics Factor Analysis (MOFA). Previous toxicology studies that integrate multiple omics layers including MOFA-based analyses to disentangle layered molecular responses in lung exposure studies^51^ demonstrate how multi-omics integration yields deeper mechanistic insights and robust methodological evaluation. Third, our exclusive use of publicly available datasets, while providing valuable insights, may limit the discovery of novel biological phenomena due to dataset biases and lack of experimental validation. Moreover, we selected A549 cells, a widely used in vitro model for respiratory toxicology, due to their well-characterized response to inhaled substances and ease of transcriptomic profiling. Nevertheless, because A549 is a single immortalized cell line, its response may not capture the full spectrum of tissue- or organism-level dynamics seen in vivo. Comparative nanotoxicology meta-analyses reported conserved responses such as oxidative stress, mitochondrial dysfunction across both fish and mammalian models. However, other responses, for instance, disrupted cysteine and methionine metabolism in *C. elegans* compared to altered ketone body synthesis in HepG2 cells. These discrepancies are partly caused by variations in the conservation and function of orthologous genes between species, which can lead to divergent molecular responses and contribute to the variability observed across studies^52^. Such biological divergence constrains the direct extrapolation of our temporal tipping points to whole organisms. Future studies should validate these dynamics in primary cells and in vivo time-course studies, ultimately integrating multi-species datasets.

Despite these limitations, this study provides an important foundation for advancing nanotoxicology research. Our analytical framework delivers significant added value by capturing the temporal evolution of stress-response pathways rather than isolated snapshots, integrating transcriptomic DEGs with pathway enrichment and PPI network topology, and enabling predictive simulations that suggest the potential tipping points leading to apoptosis under prolonged AgNPs exposure. These capabilities extend beyond conventional endpoint analyses to generate mechanistic hypotheses that can be experimentally tested and refined. The integration of transcriptomic analysis, pathway enrichment, and simulation-based approaches demonstrates the probability of reconstructing dynamic molecular responses to nanoparticle exposure. These methods offer a pathway to developing tools such as quantitative adverse outcome pathways (qAOPs), which could link molecular-level events to organism-level toxicological effects in a systematic manner. While this study does not itself establish qAOPs or predictive models for nanoparticle toxicity, it serves as a conceptual and methodological starting point for future efforts in this direction. Moreover, the dynamic reconstruction of key molecular pathways using RNA-seq data and simulation highlights the potential for transcriptomic data to inform predictive frameworks. Expanding on this foundation, integrating single-cell approaches, such as scRNA-seq, mass cytometry, and flow cytometry, provides an additional layer of granularity to nanotoxicology research. These methods reveal the intricate heterogeneity of cellular responses, enabling the identification of distinct subpopulations and their diverse reactions to nanoparticle exposure. For instance, previous study using mass cytometry have demonstrated that individual immune cells, such as B cells and naïve CD4^+^ T cells, exhibit varying sensitivities to AgNPs, responding through different stress pathways like inflammation and apoptosis^53^. Such insights underscore the critical role of cellular heterogeneity in shaping nanoparticle-induced effects, which often remain undetectable in bulk analyses. Furthermore, the integration of single-cell approaches with systems biology methodologies creates a synergistic framework for understanding nanoparticle-cell interactions at both micro- and macro-scales. Systems biology approaches provide a comprehensive perspective by capturing the dynamic evolution of cellular states over time. When combined with the granularity of single-cell data, these approaches enhance the predictive power of frameworks for nanoparticle safety assessment.

In conclusion, this work highlights the value of combining temporal analysis with computational modeling to explore the complexity of nanoparticle-induced cellular responses. While further validation and refinement are needed, this study represents a significant step toward building the foundational knowledge and tools necessary for predictive toxicology and the rational design of safer nanomaterials.

## Methods

### Publicly available RNA-seq dataset and data analysis

RNA-seq data were obtained from the ArrayExpress repository (accession number E-MTAB-5734)^33^, focusing on human lung epithelial cancer cells (A549) exposed to silver nanoparticles. AgNPs used in this study had an average core size of 15 nm and a hydrodynamic size ranging from 50 to 70 nm. A549 cells were exposed to 38.6 μg/mL of AgNPs for 1, 6, or 24 h, with four biological replicates for the control group per condition. For the AgNPs-treated group, four biological replicates were used for 1 h, and three biological replicates were used for 6, 24 h. Raw sequencing reads in FASTQ format were evaluated for quality using FastQC v0.12.0 (http://www.bioinformatics.babraham.ac.uk/projects/fastqc) to identify low-quality bases and adapter sequences. Reads were trimmed using Trimmomatic v0.39^54^, to ensure high-quality reads for downstream analysis. Trimmed reads were aligned to the human reference genome (GRCh38 assembly) using Rsubread v2.18.0 within R 4.4.0 with default parameters^55^, allowing for unique mapping of reads. Read counts were summarized from aligned reads, and differential gene expression (DGE) analysis was conducted using DESeq2 v1.44.0^56^. DEGs were identified using the Wald test, which assumes a negative binomial distribution to account for the overdispersion. Genes with a Benjamini–Hochberg adjusted *p*-value of <0.05 were classified as DEGs.

### Data visualization and functional analysis

Functional enrichment analysis of DEGs was performed using clusterProfiler v4.12.6^57,58^. Gene ontology (GO) was conducted to identify significantly over-represented biological processes. Enrichment results were considered statistically significant if the Benjamini–Hochberg adjusted *p*-value was <0.05. Visualization of enrichment results was performed using ggplot2 v3.5.2 (https://ggplot2.tidyverse.org/). Dot plots were generated to depict enriched GO terms categories, with the size of points corresponding to the number of associated genes and color indicating statistical significance. t-SNE^59^ and UMAP^60^ analyses were performed for unsupervised visualization of sample clustering based on global gene expression using Rtsne v0.17^61^ and uwot v0.2.3^62^, respectively. For t-SNE, the parameters were set to perplexity = 5. UMAP analysis was conducted with n_neighbors = 22 and min_dist = 0.1.

### Protein-protein interaction (PPI) analysis

PPI networks were constructed to identify potential interactions among the proteins encoded by DEGs. The analysis and visualization were performed using the STRINGdb v2.16.4, which integrates known and predicted protein-protein interactions^22^. Only interactions with a high confidence score (≥999) were included to ensure robust and reliable network construction. Input to the STRING database included the list of DEGs derived from DGE analysis.

### Modeling and simulation of apoptosis induced by silver nanoparticles

To elucidate the molecular responses to AgNPs exposure, a mechanistic pathway map was constructed using CellDesigner v4.4.2, a software platform for modeling and visualizing biochemical networks^23,24^. The pathway map incorporated key molecular players involved in oxidative stress, endoplasmic reticulum (ER) stress, DNA damage repair, and apoptotic signaling. The construction of the pathway was guided by DEGs derived from transcriptomic analysis and was further enriched with functional annotations from the literature. Each molecular interaction was curated to reflect known biochemical mechanisms, ensuring that the model captured the complexity of cellular stress responses. This map served as the foundation for further dynamic analyses.

### Parameter estimation and simulation of cellular stress responses and apoptosis

Dynamic simulations were performed using COPASI v4.44^25^, a biochemical simulation tool designed for analyzing reaction networks, to analyze reaction networks with irreversible mass action kinetics, representing the unidirectional nature of key biochemical processes. Initial conditions for gene expression were set using read counts normalized by the median of ratios to account for differences in sequencing depth. For the external stressor AgNPs, the initial value was fixed at 38.6, matching the nominal exposure concentration of 38.6 µg/mL in the culture medium and providing a common scaling reference for rate constant fitting. Parameter estimation for rate constants was conducted with time-course RNA-seq counts using the Levenberg-Marquardt method, with bounds set to -Infinity and +Infinity, a start value of 0.1, and other settings left as default. Simulations were run with a duration of 48 h, an interval size of 1 h, and the deterministic LSODA method. Key stress markers (*MT1/2, HMOX1, DDIT3*) were used to model the progression of oxidative stress, ER stress, and apoptotic pathways. The outcomes were analyzed to elucidate the temporal dynamics of cellular stress responses and apoptosis. The model is available from the BioModels database under accession ID MODEL2412210001.

## Supplementary information

Supplementary information

Supplementary Data 1

Supplementary Data 2

Supplementary Data 3

## Data Availability

The transcriptomic data used in this study is publicly available from ArrayExpress database under the accession number E-MTAB-5734 (https://www.ebi.ac.uk/biostudies/arrayexpress/studies/E-MTAB-5734)^33^. The models developed in this study are available on Zenodo under the identifier 10.5281/zenodo.15861077.

## Code availability

The codes for RNA-seq data processing and PPI analysis are available at https://github.com/yoon-lab/TemporalEvolution_Study.

## References

[1] Malik, S., Muhammad, K. & Waheed, Y. Nanotechnology: a revolution in modern industry. *Molecules* **28**, 661 (2023). 36677717 10.3390/molecules28020661 PMC9865684

[2] Khan, I., Saeed, K. & Khan, I. Nanoparticles: properties, applications and toxicities. *Arab. J. Chem.* **12**, 908–931 (2019).

[3] Xu, L. et al. Silver nanoparticles: synthesis, medical applications and biosafety. *Theranostics* **10**, 8996–9031 (2020). 32802176 10.7150/thno.45413 PMC7415816

[4] Zhang, X.-F., Liu, Z.-G., Shen, W. & Gurunathan, S. Silver nanoparticles: synthesis, characterization, properties, applications, and therapeutic approaches. *IJMS* **17**, 1534 (2016). 27649147 10.3390/ijms17091534 PMC5037809

[5] Abbasi, E. et al. Silver nanoparticles: synthesis methods, bio-applications and properties. *Crit. Rev. Microbiol.* 1–8, 10.3109/1040841X.2014.912200 (2014). 10.3109/1040841X.2014.912200 24937409

[6] Yu, S., Yin, Y. & Liu, J. Silver nanoparticles in the environment. *Environ. Sci. Process. Impacts* **15**, 78–92 (2013). 24592429 10.1039/c2em30595j

[7] Foldbjerg, R., Dang, D. A. & Autrup, H. Cytotoxicity and genotoxicity of silver nanoparticles in the human lung cancer cell line, A549. *Arch. Toxicol.* **85**, 743–750 (2011). 20428844 10.1007/s00204-010-0545-5

[8] Li, Y. et al. Differential genotoxicity mechanisms of silver nanoparticles and silver ions. *Arch. Toxicol.* **91**, 509–519 (2017). 27180073 10.1007/s00204-016-1730-y

[9] Fernández-Messina, L. et al. Differential mechanisms of shedding of the glycosylphosphatidylinositol (GPI)-anchored NKG2D ligands. *J. Biol. Chem.* **285**, 8543–8551 (2010). 20080967 10.1074/jbc.M109.045906 PMC2838276

[10] Gliga, A. R., Skoglund, S., Odnevall Wallinder, I., Fadeel, B. & Karlsson, H. L. Size-dependent cytotoxicity of silver nanoparticles in human lung cells: the role of cellular uptake, agglomeration and Ag release. *Part Fibre Toxicol.* **11**, 11 (2014). 24529161 10.1186/1743-8977-11-11 PMC3933429

[11] Butler, K. S., Peeler, D. J., Casey, B. J., Dair, B. J. & Elespuru, R. K. Silver nanoparticles: correlating nanoparticle size and cellular uptake with genotoxicity. *Mutagenesis* **30**, 577–591 (2015). 25964273 10.1093/mutage/gev020 PMC4566096

[12] Holmila, R. J. et al. Silver nanoparticles induce mitochondrial protein oxidation in lung cells impacting cell cycle and proliferation. *Antioxidants* **8**, 552 (2019). 31739476 10.3390/antiox8110552 PMC6912658

[13] Gliga, A. R., Di Bucchianico, S., Lindvall, J., Fadeel, B. & Karlsson, H. L. RNA-sequencing reveals long-term effects of silver nanoparticles on human lung cells. *Sci. Rep.* **8**, 6668 (2018). 29703973 10.1038/s41598-018-25085-5 PMC5923294

[14] González-Vega, J. G. et al. Lung models to evaluate silver nanoparticles’ toxicity and their impact on human health. *Nanomaterials* **12**, 2316 (2022). 35808152 10.3390/nano12132316 PMC9268743

[15] Ferdous, Z. & Nemmar, A. Health impact of silver nanoparticles: a review of the biodistribution and toxicity following various routes of exposure. *IJMS* **21**, 2375 (2020). 32235542 10.3390/ijms21072375 PMC7177798

[16] Mao, B.-H., Chen, Z.-Y., Wang, Y.-J. & Yan, S.-J. Silver nanoparticles have lethal and sublethal adverse effects on development and longevity by inducing ROS-mediated stress responses. *Sci. Rep.* **8**, 2445 (2018). 29402973 10.1038/s41598-018-20728-z PMC5799281

[17] Xu, F., Piett, C., Farkas, S., Qazzaz, M. & Syed, N. I. Silver nanoparticles (AgNPs) cause degeneration of cytoskeleton and disrupt synaptic machinery of cultured cortical neurons. *Mol. Brain* **6**, 29 (2013). 23782671 10.1186/1756-6606-6-29 PMC3695839

[18] Li, J. et al. Silver nanoparticles modulate mitochondrial dynamics and biogenesis in HepG2 cells. *Environ. Pollut.* **256**, 113430 (2020). 31685329 10.1016/j.envpol.2019.113430

[19] Ferdous, Z., Al-Salam, S., Greish, Y. E., Ali, B. H. & Nemmar, A. Pulmonary exposure to silver nanoparticles impairs cardiovascular homeostasis: effects of coating, dose and time. *Toxicol. Appl. Pharmacol.* **367**, 36–50 (2019). 30639276 10.1016/j.taap.2019.01.006

[20] Kim, S. & Ryu, D. Silver nanoparticle-induced oxidative stress, genotoxicity and apoptosis in cultured cells and animal tissues. *J. Appl. Toxicol.* **33**, 78–89 (2013). 22936301 10.1002/jat.2792

[21] Tabandeh, M. R., Samie, K. A., Mobarakeh, E. S., Khadem, M. D. & Jozaie, S. Silver nanoparticles induce oxidative stress, apoptosis and impaired steroidogenesis in ovarian granulosa cells of cattle. *Anim. Reprod. Sci.* **236**, 106908 (2022). 34920187 10.1016/j.anireprosci.2021.106908

[22] Szklarczyk, D. et al. The STRING database in 2023: protein–protein association networks and functional enrichment analyses for any sequenced genome of interest. *Nucleic Acids Res.* **51**, D638–D646 (2023). 36370105 10.1093/nar/gkac1000 PMC9825434

[23] Funahashi, A. et al. CellDesigner 3.5: a versatile modeling tool for biochemical networks. *Proc. IEEE* **96**, 1254–1265 (2008).

[24] Funahashi, A., Morohashi, M., Kitano, H. & Tanimura, N. CellDesigner: a process diagram editor for gene-regulatory and biochemical networks. *BIOSILICO* **1**, 159–162 (2003).

[25] Hoops, S. et al. COPASI—a COmplex PAthway SImulator. *Bioinformatics* **22**, 3067–3074 (2006). 17032683 10.1093/bioinformatics/btl485

[26] Li, D., Ding, Z., Du, K., Ye, X. & Cheng, S. Reactive oxygen species as a link between antioxidant pathways and autophagy. *Oxid. Med. Cell. Longev.* **2021**, 5583215 (2021). 34336103 10.1155/2021/5583215 PMC8324391

[27] Bhattarai, K. R., Riaz, T. A., Kim, H.-R. & Chae, H.-J. The aftermath of the interplay between the endoplasmic reticulum stress response and redox signaling. *Exp. Mol. Med.* **53**, 151–167 (2021). 33558590 10.1038/s12276-021-00560-8 PMC8080639

[28] Chen, X., Shi, C., He, M., Xiong, S. & Xia, X. Endoplasmic reticulum stress: molecular mechanism and therapeutic targets. *Sig. Transduct. Target Ther.* **8**, 352 (2023). 10.1038/s41392-023-01570-w PMC10502142 37709773

[29] Aubrey, B. J., Kelly, G. L., Janic, A., Herold, M. J. & Strasser, A. How does p53 induce apoptosis and how does this relate to p53-mediated tumour suppression?. *Cell Death Differ.* **25**, 104–113 (2018). 29149101 10.1038/cdd.2017.169 PMC5729529

[30] Liebl, M. C. & Hofmann, T. G. Cell fate regulation upon DNA damage: p53 serine 46 kinases pave the cell death road. *BioEssays* **41**, 1900127 (2019). 10.1002/bies.201900127 31621101

[31] Reyes, J. et al. Fluctuations in p53 signaling allow escape from cell-cycle arrest. *Mol. Cell* **71**, 581–591.e5 (2018). 30057196 10.1016/j.molcel.2018.06.031 PMC6282757

[32] Elmore, S. Apoptosis: a review of programmed cell death. *Toxicol. Pathol.* **35**, 495–516 (2007). 17562483 10.1080/01926230701320337 PMC2117903

[33] Dekkers, S. et al. Multi-omics approaches confirm metal ions mediate the main toxicological pathways of metal-bearing nanoparticles in lung epithelial A549 cells. *Environ. Sci. Nano* **5**, 1506–1517 (2018).

[34] Mikhailova, E. O. Silver nanoparticles: mechanism of action and probable bio-application. *JFB* **11**, 84 (2020). 33255874 10.3390/jfb11040084 PMC7711612

[35] Baltaci, A. K., Yuce, K. & Mogulkoc, R. Zinc metabolism and metallothioneins. *Biol. Trace Elem. Res.* **183**, 22–31 (2018). 28812260 10.1007/s12011-017-1119-7

[36] Günther, V., Lindert, U. & Schaffner, W. The taste of heavy metals: gene regulation by MTF-1. *Biochim. Biophys. Acta Mol. Cell Res.* **1823**, 1416–1425 (2012). 10.1016/j.bbamcr.2012.01.005 22289350

[37] Heuchel, R. et al. The transcription factor MTF-1 is essential for basal and heavy metal-induced metallothionein gene expression. *EMBO J.* **13**, 2870–2875 (1994). 8026472 10.1002/j.1460-2075.1994.tb06581.x PMC395168

[38] Rice, J. M., Zweifach, A. & Lynes, M. A. Metallothionein regulates intracellular zinc signaling during CD4+ T cell activation. *BMC Immunol.* **17**, 13 (2016). 27251638 10.1186/s12865-016-0151-2 PMC4890327

[39] Chiang, S.-K., Chen, S.-E. & Chang, L.-C. The Role of HO-1 and its crosstalk with oxidative stress in cancer cell survival. *Cells* **10**, 2401 (2021). 34572050 10.3390/cells10092401 PMC8471703

[40] Gozzelino, R., Jeney, V. & Soares, M. P. Mechanisms of cell protection by heme oxygenase-1. *Annu. Rev. Pharmacol. Toxicol.* **50**, 323–354 (2010). 20055707 10.1146/annurev.pharmtox.010909.105600

[41] Kang, S. J., Ryoo, I., Lee, Y. J. & Kwak, M.-K. Role of the Nrf2-heme oxygenase-1 pathway in silver nanoparticle-mediated cytotoxicity. *Toxicol. Appl. Pharmacol.* **258**, 89–98 (2012). 22036727 10.1016/j.taap.2011.10.011

[42] Oslowski, C. M. & Urano, F. Measuring ER stress and the unfolded protein response using mammalian tissue culture system. In *Methods in Enzymology* vol. 490 71–92 (Elsevier, 2011). 10.1016/B978-0-12-385114-7.00004-0 PMC3701721 21266244

[43] Seo, Y.-W. et al. The molecular mechanism of Noxa-induced mitochondrial dysfunction in p53-mediated cell death. *J. Biol. Chem.* **278**, 48292–48299 (2003). 14500711 10.1074/jbc.M308785200

[44] Sun, Y. & Leaman, D. W. Involvement of Noxa in cellular apoptotic responses to interferon, double-stranded RNA, and virus infection. *J. Biol. Chem.* **280**, 15561–15568 (2005). 15705586 10.1074/jbc.M412630200

[45] Chen, L. N., Wang, Y., Ma, D. L. & Chen, Y. Y. Short interfering RNA against the PDCD5 attenuates cell apoptosis and caspase-3 activity induced by Bax overexpression. *Apoptosis* **11**, 101–111 (2006). 16374546 10.1007/s10495-005-3134-y

[46] Li, G., Ma, D. & Chen, Y. Cellular functions of programmed cell death 5. *Biochim. Biophys. Acta Mol. Cell Res.* **1863**, 572–580 (2016). 10.1016/j.bbamcr.2015.12.021 26775586

[47] Hanna, J., Guerra-Moreno, A., Ang, J. & Micoogullari, Y. Protein degradation and the pathologic basis of disease. *Am. J. Pathol.* **189**, 94–103 (2019). 30312581 10.1016/j.ajpath.2018.09.004 PMC6315326

[48] Sass, S. et al. MicroRNAs coordinately regulate protein complexes. *BMC Syst. Biol.* **5**, 136 (2011). 21867514 10.1186/1752-0509-5-136 PMC3170341

[49] Agarwal, A. et al. Potential biological role of poly (ADP-ribose) polymerase (PARP) in male gametes. *Reprod. Biol. Endocrinol.* **7**, 143 (2009). 19961617 10.1186/1477-7827-7-143 PMC2800114

[50] Zhong, Q. et al. Protein posttranslational modifications in health and diseases: functions, regulatory mechanisms, and therapeutic implications. *MedComm* **4**, e261 (2023). 37143582 10.1002/mco2.261 PMC10152985

[51] Titz, B. et al. Multi-omics systems toxicology study of mouse lung assessing the effects of aerosols from two heat-not-burn tobacco products and cigarette smoke. *Computational Struct. Biotechnol. J.* **18**, 1056–1073 (2020). 10.1016/j.csbj.2020.04.011 PMC7218232 32419906

[52] Anh, N. H. et al. Unveiling potentially convergent key events related to adverse outcome pathways induced by silver nanoparticles via cross-species omics-scale analysis. *J. Hazard. Mater.* **459**, 132208 (2023). 37544172 10.1016/j.jhazmat.2023.132208

[53] Ha, M. K. et al. Mass cytometry and single-cell RNA-seq profiling of the heterogeneity in human peripheral blood mononuclear cells interacting with silver nanoparticles. *Small* **16**, 1907674 (2020). 10.1002/smll.201907674 32163679

[54] Bolger, A. M., Lohse, M. & Usadel, B. Trimmomatic: a flexible trimmer for Illumina sequence data. *Bioinformatics* **30**, 2114–2120 (2014). 24695404 10.1093/bioinformatics/btu170 PMC4103590

[55] Liao, Y., Smyth, G. K. & Shi, W. The R package Rsubread is easier, faster, cheaper and better for alignment and quantification of RNA sequencing reads. *Nucleic Acids Res.* **47**, e47–e47 (2019). 30783653 10.1093/nar/gkz114 PMC6486549

[56] Love, M. I., Huber, W. & Anders, S. Moderated estimation of fold change and dispersion for RNA-seq data with DESeq2. *Genome Biol.* **15**, 550 (2014). 25516281 10.1186/s13059-014-0550-8 PMC4302049

[57] Wu, T. et al. clusterProfiler 4.0: A universal enrichment tool for interpreting omics data. *Innovation* **2**, 100141 (2021). 34557778 10.1016/j.xinn.2021.100141 PMC8454663

[58] Yu, G., Wang, L.-G., Han, Y. & He, Q.-Y. clusterProfiler: an R package for comparing biological themes among gene clusters. *OMICS J. Integr. Biol.* **16**, 284–287 (2012). 10.1089/omi.2011.0118 PMC3339379 22455463

[59] Van Der Maaten, L. & Hinton, G. Visualizing data using t-SNE. *J. Mach. Learn. Res.* **9**, 2579–2605 (2008).

[60] McInnes, L., Healy, J., Saul, N. & Großberger, L. UMAP: uniform manifold approximation and projection. *JOSS* **3**, 861 (2018).

[61] Krijthe, J. Rtsne: T-Distributed Stochastic Neighbor Embedding using a Barnes-Hut Implementation. 0.17 10.32614/CRAN.package.Rtsne (2014).

[62] Melville, J. uwot: The Uniform Manifold Approximation and Projection (UMAP) Method for Dimensionality Reduction. 0.2.3 10.32614/CRAN.package.uwot (2019).

