# Supplementary material for: A systems biology approach to understand temporal evolution of silver nanoparticle toxicity
Source: NPJ Syst Biol Appl. 2025 Jul 19;11:80. doi: 10.1038/s41540-025-00561-7 (PMC12274429; doi:10.1038/s41540-025-00561-7)
Supplement: Supplementary file 1 — Supplementary information [file 41540_2025_561_MOESM1_ESM.docx]

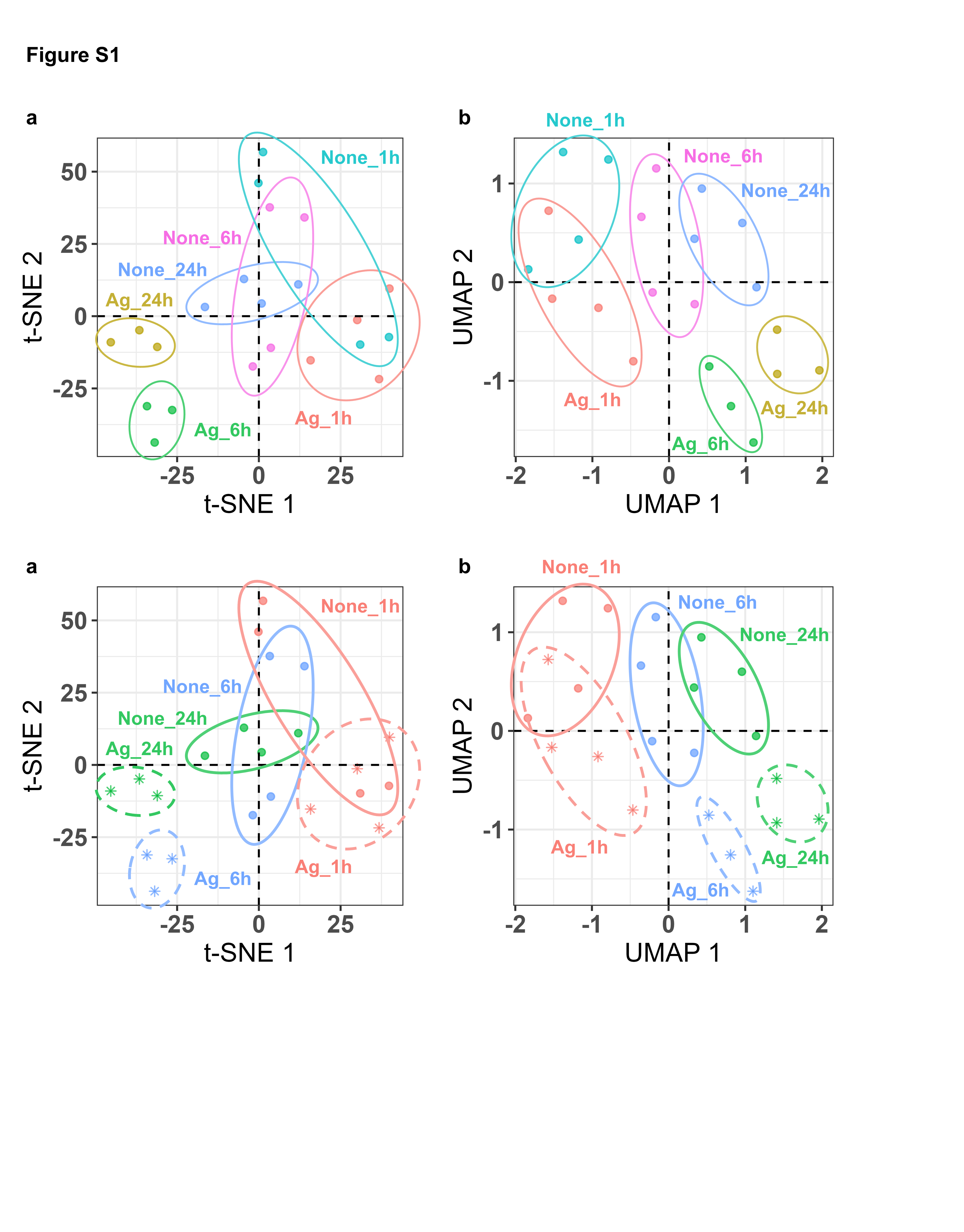


**Fig. S1. Visualization of gene expression profiles in control and AgNPs-treated samples across time points using dimensionality reduction techniques.**

a) t-SNE and b) UMAP projections of gene expression profiles in AgNPs-treated and control samples at 1 h, 6 h, and 24 h are represented by red, blue and green, respectively. Sample groups are distinguished by shape: solid circles for control and asterisks for AgNPs-treated samples.
